# Lights and Shadows of a Primary School-Based Nutrition Education Program in Italy: Insights from the LIVELY Project

**DOI:** 10.3390/nu17172778

**Published:** 2025-08-27

**Authors:** Sara Basilico, Maria Vittoria Conti, Ilaria Ardoino, Chiara Breda, Federica Loperfido, Francesca Orsini, Maria Luisa Ojeda Fernandez, Laura Pierini, Stefano Conca Bonizzoni, Elisabetta Modena, Federica Villa, Hellas Cena, Marta Baviera, Carlotta Franchi

**Affiliations:** 1 Laboratory of Dietetics and Clinical Nutrition, Department of Public Health, Experimental and Forensic Medicine, University of Pavia, 27100 Pavia, Italy; mariavittoria.conti@unipv.it (M.V.C.); chiara.breda@unipv.it (C.B.); federica.loperfido@unipv.it (F.L.); hellas.cena@unipv.it (H.C.); 2 Laboratory of Pharmacoepidemiology and Human Nutrition, Department of Health Policy, Istituto di Ricerche Farmacologiche Mario Negri IRCCS, 20156 Milan, Italy; ilaria.ardoino@marionegri.it (I.A.); francesca.orsini@marionegri.it (F.O.); laura.pierini@marionegri.it (L.P.); carlotta.franchi@marionegri.it (C.F.); 3 Laboratory of Cardiovascular Prevention, Istituto di Ricerche Farmacologiche Mario Negri IRCCS, 20156 Milan, Italy; luisa.ojeda@marionegri.it (M.L.O.F.); marta.baviera@marionegri.it (M.B.); 4 Department of Humanities, University of Pavia, 27100 Pavia, Italy; stefano.concabonizzoni@unipv.it (S.C.B.); elisabetta.modena@unipv.it (E.M.); federica.villa@unipv.it (F.V.); 5 Clinical Nutrition Unit, ICS Maugeri, Istituti di Ricovero e Cura a Carattere Scientifico (IRCCS), 27100 Pavia, Italy; 6 Italian Institute for Planetary Health, 20156 Milan, Italy

**Keywords:** childhood obesity, healthy lifestyle, primary prevention, nutritional education, mediterranean diet

## Abstract

**Background/Objectives**: Childhood obesity represents a pressing global health challenge, demanding coordinated, long-term strategies. Schools and families are pivotal environments for shaping children’s lifestyle behaviors. The LIVELY project aimed to assess overweight/obesity prevalence and associated factors in primary school children, and to implement a multidimensional educational intervention promoting healthy, sustainable lifestyles. **Methods**: This single-arm study was conducted from October 2023 to October 2024 in a primary school in Milan. The intervention included age and culturally tailored lessons, games, and activities. Anthropometric measures, dietary adherence, and lifestyle habits were assessed before and after the intervention. Satisfaction surveys were administered to children, families, and teachers. **Results**: The project involved 227 children across 14 classes (mean age 8.9, SD 1.2 years). The prevalence of overweight/obesity was 23.4%. Adherence to the Mediterranean Diet was moderate, limited by low intake of vegetables, nuts, and dairy. Physical activity was low, screen time excessive, and sleep insufficient. No statistically significant improvements in anthropometrics or diet adherence were observed post-intervention, but positive trends emerged for physical activity, sleep, and hydration. Over half of the children passed the nutritional knowledge test. Despite these challenges, high satisfaction levels have been shared by children, parents, and teachers. **Conclusions**: The limited duration of the intervention and challenges engaging families in a low socio-economic context may have constrained the impact of the program, and caution is advised in generalizing the findings. The LIVELY project highlights the complexity of tackling childhood obesity in multicultural settings and emphasizes the need for longer, continuous, and culturally tailored programs that actively involve families to promote sustainable healthy behaviors.

## 1. Introduction

Childhood obesity is recognized as a chronic and multifactorial disease, influenced by a combination of genetic, environmental, and behavioral factors [1]. Over the past decades, the prevalence of childhood obesity has increased significantly worldwide, representing one of the most pressing public health challenges [2]. Children with obesity are at higher risk of developing metabolic disorders, cardiovascular diseases, and psychological complications, with long-term consequences that may persist into adulthood [3,4]. Therefore, the prevention of childhood obesity requires comprehensive and timely intervention strategies that address both individual behaviors and broader environmental influences [5,6].

According to the most recent report from the World Health Organization (WHO) for the European region, the prevalence of overweight (including obesity) among children aged 7 to 9 years is 25% in countries participating in the sixth round of the Childhood Obesity Surveillance Initiative (COSI) [7]. In Italy, despite efforts made in recent years, childhood overweight and obesity rates remain alarming. Italy ranks second in Europe for childhood overweight and obesity prevalence, with rates just below 40%, surpassed only by Cyprus and followed by Greece and Croatia [7]. In Italy, territorial differences reveal a North–South divide, with children living in the South showing almost three times the prevalence of those living in the North, and double that of those residents in the central regions, with a similar gap among adults [8,9].

One of the key contributors to the rising obesity rates is the ongoing global nutritional transition, characterized by a shift from traditional dietary patterns, such as the Mediterranean Diet (MD), to Westernized diets rich in ultra-processed foods, refined sugars, and unhealthy fats [10,11]. This trend, initially observed in high-income countries, has now spread to low- and middle-income regions, exacerbating the burden of obesity and related non-communicable diseases [12]. Simultaneously, modern lifestyles have become increasingly sedentary, with children spending more time engaging in screen-based activities and less in physical exercise [13,14,15]. The widespread use of electronic devices has significantly increased screen time, further reducing opportunities for active play and structured physical activity [16]. This shift has been linked to a higher risk of overweight and obesity, as well as negative effects on mental health and academic performance [17,18,19].

Adding complexity to this scenario is the growing multicultural composition of school populations, driven by increased migration flows. In many urban settings, including Italy, primary school now reflects a diverse mix of cultural backgrounds, socioeconomic statuses, and dietary habits [20]. Such diversity influences children’s nutritional behaviors and their families’ food choices, often creating additional barriers to achieving a balanced diet [21,22,23].

Research suggests that children from lower-income and migrant families may have limited access to healthy foods, increased exposure to obesogenic environments, and undergo cultural shifts toward energy-dense, nutrient-poor dietary patterns [24,25]. Therefore, school-based interventions must address these socio-cultural determinants to ensure inclusivity and effectiveness. Nutritional education is widely recognized as a key tool to promote healthy eating habits and prevent obesity [26,27]. Both developed and developing countries have increasingly adopted school-based nutrition education programs aimed at improving children’s dietary behaviors and overall health [28,29]. Notably, the “Eat Healthy, Grow Healthy” project in Ghana, a low-middle-income country, demonstrated significant improvements in children’s nutrition-related knowledge and fruit consumption. Similarly, a food education intervention in the Republic of Palau (Oceania) resulted in enhanced nutritional knowledge and increased intake of fruits and vegetables [30]. Additionally, a program implemented in Malaysia showed promising outcomes in improving children’s eating habits and physical activity levels [31]. These examples highlight the potential effectiveness of culturally tailored nutrition education interventions across diverse socioeconomic contexts.

In Italy, the Ministry of Education, University, and Research (MIUR) has promoted initiatives to integrate nutritional education into school curricula; however, this is not yet a standardized nationwide initiative. [32,33]. Among notable recent interventions, the EAT project evaluated a school-based multicomponent educational program in middle-school adolescents, demonstrating significant improvements in adiposity measures and eating behaviors [34]. Other initiatives targeting younger children include the “Maestra Natura” project, led by the National Institute of Health and the Ministry of Health, which significantly improved nutritional knowledge in primary school children [32,35], and the “Giochiamo” project, which combined education and play to enhance nutrition and physical activity behaviors in 6–8-year-olds [36]. Additionally, Italy participates in the EU’s “School Fruit and Vegetables Scheme” (“Frutta nelle Scuole”) to encourage fruit and vegetable consumption among students [33].

In this context, the LIVELY project (MuLtidimensional school-based and family Involved interVentions, to promote a hEalthy and sustainable LifestYle for the childhood obesity primary prevention) was developed to address childhood obesity through a multidimensional approach that integrates school-based nutritional education with family involvement.

Through the implementation of an engaging and interactive educational curriculum, the LIVELY study sought to enhance children’s knowledge of healthy eating and sustainable food choices, fostering an environment conducive to long-term behavioral change. The ultimate goal was to provide a replicable model for integrating nutritional education into school curricula, reinforcing the role of schools as key players in obesity prevention strategies.

## 2. Materials and Methods

### 2.1. Study Design and Setting

**Figure 1:**
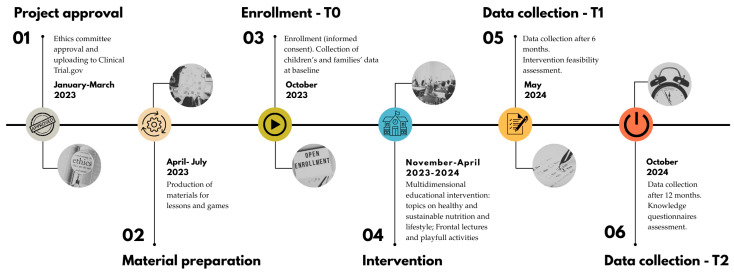
Timeline of the LIVELY project.

The LIVELY project was a single-arm pre-post study (without control) conducted by a multidisciplinary team consisting of researchers from the Istituto di Ricerche Farmacologiche Mario Negri IRCCS (IMN) in Milan and the Laboratory of Dietetics and Clinical Nutrition (LDNC) at the University of Pavia [37]. The study was carried out at Istituto Comprensivo “Luigi Cadorna”, a public primary school with a strong multiethnic component in the North–West of Milan, Italy, from October 2023 to October 2024. The study was approved by the Fondazione IRCCS Istituto Neurologico Carlo Besta Ethics Committee (protocol 11) in January 2023. The timeline of the study is shown in Figure 1.

### 2.2. Study Objectives

The primary objective of this study was to assess the prevalence of overweight and obesity among primary school children and to identify the key determinants related to their lifestyle habits, family socioeconomic status, and environmental influences.

The secondary objective was to evaluate the feasibility of a structured multidimensional educational intervention in terms of participant satisfaction, learning outcomes, and organizational impact within the school environment. Furthermore, the project aimed to examine potential changes in children’s dietary and lifestyle habits, as well as shifts in family behavioral attitudes, at 6 and 12 months post-intervention.

### 2.3. Selection Criteria and Enrollment

The target population was children from 5 to 12 years of age, of both sexes and any ethnicity, without any exclusion criteria.

In June 2023, during the final teaching board meeting, the researchers presented the project to the dean and teachers. The project was approved by 14 classes, which subsequently included it in the curriculum for the 2023–2024 academic year.

In October 2023, an informed consent and privacy form was distributed to the parents of the children in the participating classes. Participation in the project was voluntary, and only the children whose parents returned the signed consent form were enrolled in the study and involved in the data collection (anthropometric measurements and questionnaires). However, as the educational intervention was integrated in a mandatory curricular subject (Civic Education) and took place during school hours from November 2023 to April 2024, all children attending the participating classes were assessable for the evaluation of learning and of the appreciation of the project, regardless of their inclusion in the data collection.

### 2.4. Educational Intervention

The educational intervention was structured into six modules, interspersed with a multimedia workshop: details about the six modules are provided in Table 1. Each lesson, held every four weeks and lasting two hours, was divided into two parts: the first part consisted of approximately 30–40 min of frontal teaching supported by interactive slides, followed by a second part of about 80–90 min, during which playful activities tailored to the children’s age group were carried out. The educational intervention was conducted by experienced trained professionals, including nutritional biologists and registered dietitians. The teaching materials were developed based on the 2018 Centro di Ricerca Alimenti e Nutrizione (CREA) Guidelines (“*Linee guida per una sana alimentazione*”) [38], the WHO Guidelines [39], and the Harvard Kids’ Healthy Eating Plate [40].

After the first five lessons, in which children learned the fundamentals of healthy and sustainable eating, a multimedia workshop was carried out in collaboration with Officine Creative of the University of Pavia. The aim of the workshop was to build upon the content learned in previous lessons to encourage discussion on healthy eating among students, develop character design, and create shared dramaturgies.

#### 2.4.1. Multimedia Workshop

**Figure 2:**
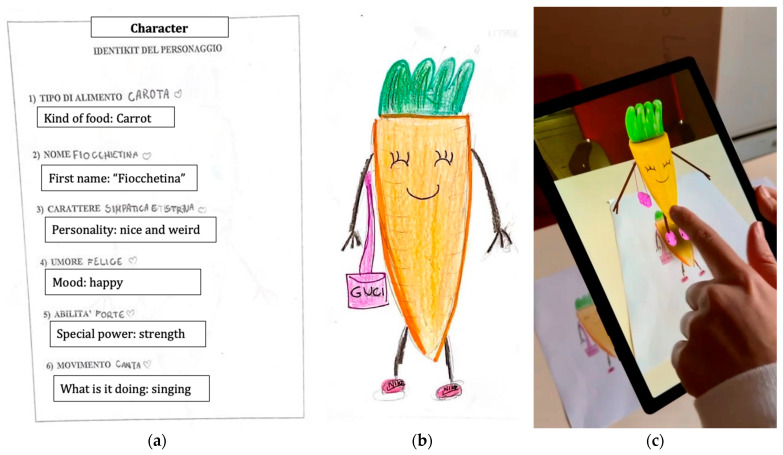
Example of a character realized during the multimedia workshop and animated using augmented reality techniques. (**a**) Description of the main features of the depicted fantasy character; (**b**) Humanized carrot drawn by a fourth-grade class; (**c**) Animation through a tablet.

During the multimedia workshop, the children were divided into small groups of approximately 5–6 participants. Each group took turns drawing a food item from a bag, relying solely on touch to identify it. The selected foods could be either fresh or packaged. After all the groups had made their selection, the entire class voted and chose a single food item to work on. At this stage, each group created a character based on the chosen food item by filling out a character profile, assigning it a name, personality traits, mood, special abilities, and a distinctive action (e.g., dancing or singing) (Figure 2a). Using this profile as a reference, the children then illustrated their food item in a humanized form (Figure 2b).

Next, four children were called to roll the “story-creator” cubes, which determined key elements of the narrative: the first cube set the location, the second introduced a helper, the third revealed a magical object, and the fourth introduced an unexpected event. Each group then crafted a story incorporating these elements along with their humanized food character as the protagonist.

Finally, five of the drawings were selected, processed, and animated using augmented reality techniques (Figure 2c).

#### 2.4.2. Family Engagement

Families were involved throughout the entire project. At the end of each lesson, to enhance family involvement and reinforce learning at home, a summary slide of the class content was shared with the parents (Figure S1). To further enhance family engagement, a multicultural celebration was held at the end of the school year (T1). Each family was invited to bring a traditional dish from their country of origin. During the event, two deliverables created during the year were distributed.

The first was a feature: on one side, the healthy plate model presented during the lessons (Module 5), and on the other side, tips for preparing a complete and balanced breakfast (Module 6) (Figure S2).

Additionally, during the school year, children were asked to write down a healthy traditional recipe from their country of origin, with the help of their parents. All the recipes were collected, categorized (appetizers, main courses, side dishes, desserts), and compiled into a PDF recipe book that was distributed during the final event. For each recipe, a brief comment was added, highlighting its nutritional value or providing a tip to make it healthier and more sustainable [https://backend.onfoods.it//sites/default/files/2024-06/Ricettario%20multietnico%20Progetto%20LIVELY.pdf (Accesed on 27 August 2025)].

### 2.5. Study Data Collection

Referring teachers distributed an ad hoc Case Report Form (CRF), in paper format, to each child whose parents signed the informed consent, and then took charge of collecting them once they were filled out by the parents. The questionnaires were translated into different languages (Italian, Arabic, and English) due to the high multiethnicity of the school.

Researchers at the IMN reported the data into an electronic, web-based CRF (eCRF) implemented using the REDCap software platform and specifically made for this study [41]. Each participant was assigned a unique code to link their data securely while protecting their identity.

Anthropometric measures and all the other questionnaires were collected at baseline (October 2023, T0), at 6 months (May 2024, T1), and at 12 months after intervention delivery (October 2024, T2).

### 2.6. Primary Outcome

#### 2.6.1. Anthropometric Measures Assessment 

Children’s anthropometric measurements were assessed by a team of trained nutritionists.

Children’s weight was measured using the same digital scale (Tanita BC545N), and it was recorded to the nearest 0.1 kg. Height was measured using a portable altimeter (Seca 213) and recorded to the nearest millimeter. All children were measured without shoes and wearing light clothing. In addition, the type of clothing worn at the time of measurement was recorded for each child. Waist circumference (WC) was measured with an inelastic tape placed midway between the lower rib and the iliac crest and recorded to the closest 0.5 cm. The measurement was taken while the child was standing upright, with feet together and arms hanging down at the sides. The circumference of the non-dominant bicep was measured with an inelastic tape placed between the acromion process and the olecranon with the forearm flexed 90°, and recorded to the closest 1.0 cm

Body Mass Index (BMI) was calculated as kg/m^2^ and expressed as BMI z-score by referring to the growth curves by sex and age proposed by the WHO that classify children as having underweight (BMI −2 standard deviation, [SD], below the median BMI) normal weight (BMI between −2 and +1 SD), overweight (BMI +1 SD, above the median BMI, equivalent to BMI 25 kg/m^2^ at 19 years) or obesity (BMI +2 SD, equivalent to BMI 30 kg/m^2^ at 19 years) [42]. The software Anthro^®^ and AnthroPlus^®^ were used to calculate the BMI z-score [43]. In addition, the waist-to-height ratio (WHtR) was calculated as waist circumference divided by height, both expressed in centimeters. For the definition of central distribution of adiposity, the discriminatory value of 0.5 for WHtR was adopted [44,45]. Children were divided into two groups: the first one included children with WHtR < 0.5 (normal distribution of adiposity), while the second one included children with WHtR ≥ 0.5 (central obesity).

#### 2.6.2. Sociodemographic Information Assessment 

Data concerning the families (household composition, ethnicity, parents’ education, and lifestyle) and the children (age, attended class, health conditions, and food habits within the family) were collected through a structured questionnaire. The latter included information about whether meals were consumed with family members, the use of electronic devices (TV or smartphones) during meals, the availability and use of sweets (e.g., as rewards), and the frequency of fast food consumption.

#### 2.6.3. Eating Behaviors and Lifestyle Assessment

Specifically, the KIDMED questionnaire (Mediterranean Diet Quality Index for children and adolescents) [46,47] was used to assess adherence to the Mediterranean Diet (MD) and to obtain an overview of the child’s consumption of the main food groups (fruits, vegetables, pasta and cereals, legumes, fish, meat, and dairy products). Adherence to MD was rated as low (score ≤ 4), medium (score between 5 and 7), and high (score ≥ 8). To complete the questionnaire, information was requested regarding the number of meals consumed, whether children ate in the school cafeteria, whether they used sweeteners in tea or milk (e.g., sweetened cocoa, sugar, or honey), whether they consumed whole foods, and whether they made consumption of ultra-processed foods (NOVA classification) [48,49].

In addition, children’s hydration levels were assessed based on the 2018 Centro di Ricerca Alimenti e Nutrizione (CREA) Guidelines, while type and load of physical activity, sleep habits, and screen time were assessed following WHO Guidelines [38,39].

### 2.7. Secondary Outcome

#### 2.7.1. Evaluation of the Educational Intervention

The feasibility of the multidimensional educational intervention at school was assessed through an ad hoc questionnaire at T1, in terms of “satisfaction” (by children, families, and teachers) and “organizational” impact in the work context (by teachers). The overall satisfaction and organizational impact of the interventions explored the following issues: usefulness (definition and consistency/congruence of the multidimensional interventions with the project objectives, usefulness of the interventions and materials prepared), didactics (competence and appropriateness of the teaching techniques of the study teams addressed to the subjects, support received by the study teams during the implementation of the activities), organization and services (timing, facilities and appropriateness of the schools’ premises). In addition, teachers were asked to refer to the reproducibility of the intervention in other classrooms or schools. All items in the questionnaire were rated on a Likert-type scale.

#### 2.7.2. Evaluation of Children’s Knowledge

The assessment of children’s knowledge on the topics covered during the nutrition education intervention was conducted using a structured questionnaire at T2. This questionnaire included true/false questions, multiple-choice items, and fill-in-the-blank exercises, with specific questions assigned to each topic. For each topic, children received a score from 1 to 10; the final score was calculated as the sum of the scores across these six topics, with a maximum possible score of 60. A passing score was defined as correctly answering at least 60% of the questions, equivalent to a minimum score of 36. This threshold was chosen based on common educational standards, where 60% is typically considered the minimum level of basic competence. It reflects a sufficient understanding of the key concepts delivered during the intervention while allowing for some margin of error, especially given the young age of the participants and the novelty of some of the content.

### 2.8. Statistical Methods

#### 2.8.1. Sample Size Calculation

We expected to enroll about 300 to 350 children aged between 6 and 12 years from 15 to 20 classes (in primary school and in the first class of secondary school), based on the availability and willingness of teachers to participate, and the resources [37]. This would have allowed us to estimate the prevalence of overweight and obesity between 23% and 24%, with a 20% relative precision and within a 95% CI [37]. These data are in line with those observed in the Okkio alla Salute 2019 survey in the Lombardy region [9].

#### 2.8.2. Statistical Analysis

Continuous variables were expressed as mean and SD or median and interquartile range (IQR) as appropriate, and categorical variables as frequencies and percentages. Continuous variables were compared using a T-test, and categorical variables were compared with a Chi-square test. Main determinants of overweight/obesity among children have been investigated among dietary patterns and lifestyle habits, evaluated at the baseline, by means of logistic regression models. Two separate models have been fitted: the first adjusted for sex, ethnic group, and dietary habits (i.e., adherence to MD and hydration), the second adjusted for sex, ethnic group, and lifestyle habits (i.e., physical activity, screen time, and sleeping habits). Results were expressed as odds ratios (ORs) and corresponding 95% confidence intervals (95% CIs). Changes in dietary and lifestyle habits in children, behavioral attitudes in their families, and children’s anthropometric measurements have been assessed at 6 months, starting from baseline. However, it was not possible to evaluate potential changes at 12 months due to the substantial reduction in sample size and the presence of attrition bias. To investigate the appreciation of the interventions, the frequencies of Likert scores for each item of the questionnaires have been calculated, and the distributions are presented according to their frequency.

## 3. Results

Fourteen classes (one first grade, five third grade, two fourth grade, and six fifth grade) with a total of 275 children, joined the educational intervention. No first-grade class took part in it.

**Figure 3:**
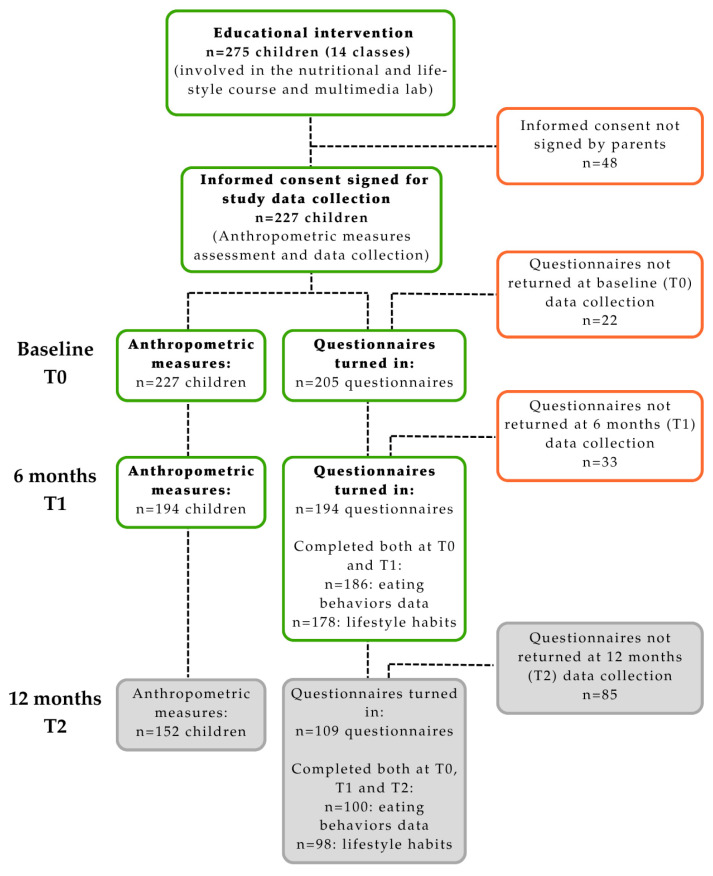
Flowchart of the sample.

Of these, 227 children, whose parents approved and signed the informed consent, were enrolled in the study data collection. Finally, 205 children returned the questionnaires and were included in the analyses. Figure 3 shows the flowchart of the study.

### 3.1. Demographic and Anthropometric Characteristics

Main characteristics of the 227 children enrolled are shown in Table 2. The mean (SD) age was 8.9 years (1.2 years), and 48% (*n* = 110) were males.

At baseline (T0), among the overall sample, 41 children (18.1%, 95% CI: 13.3–23.7%) presented overweight, while 12 (5.3%, 95% CI: 2.8–9.1%) presented obesity. Prevalence of overweight (including obesity) was almost similar between males and females (23.6% vs. 23.1%); instead, males had a higher prevalence of obesity than females (9.1% vs. 1.7%, *p* = 0.03, respectively).

Differences among ethnic groups were observed: the highest prevalence of overweight was observed among Hispanic children (*n* = 9, 29.0%), followed by African children (*n* = 21, 24.4%), while the lowest was among Caucasian (*n* = 6, 7.3%), although obesity was almost similar among them (Table S1). The overall prevalence of central obesity was 33.9%, with no significant difference between males and females (30.9% vs. 36.8%). Difference exists among ethnic groups, with Hispanic children showing the highest prevalence (58.1%, respectively, vs. Caucasic 24.4%) (Table S1).

By comparing children’s measured BMI categories with parents’ perception of their child’s weight status (underweight, normal weight, overweight), it was found that nearly 60% of parents classified their child’s weight status correctly. However, among children with overweight or obesity, parents were much more likely to underestimate their child’s condition, doing so in 80.6% of cases for children with overweight and 90.0% of cases for children with obesity.

Among parents, the prevalence of overweight and obesity was notably high, with 168 (82.0%) children having at least one parent presenting overweight (including obesity): 37.8% of mothers and 50.3% of fathers with overweight, and 17.6% of mothers and 10% of fathers with obesity. Additional data related to parents are presented in Supplementary Table S2.

### 3.2. Eating Behaviors and Lifestyle Habits

Data regarding eating behaviors and lifestyle habits at baseline are reported in Table 3. Out of 205 children with assessable data, most children (n = 111, 55.2%) are moderately adherent to MD; only 29 (14.4%) were poorly adherent. The low adherence was mainly driven by eating few vegetables (n = 122, 61.0%), not eating enough nuts (n = 138, 68.3%), and dairy food (n = 140, 69.6%) (Table S3). No significant difference emerged among ethnic groups, nor among sex, although a higher frequency of males showed good adherence to MD with respect to females (36.4% vs. 24.5%). The most consumed ultra-processed foods (more than 4 times per week) were biscuits (44.3.0%), followed by fruit juices (37.5%), and milk-based beverages (35.9%) (Table S4).

Regarding physical activity, 9% of the children did not engage in any physical activity, while 93 (46%) and 40 (20%) reported performing a sport 1–2 or 3 times or more per week, respectively, with a median of 2 h/week (IQR: 2–3), without addressing the WHO recommendations.

Regarding screen time, nearly 11% of the respondents’ children spent 3 or more hours per day in front of a screen. As for sleep duration, most of the sample (67%) met the recommended guidelines by sleeping more than 9 h per night.

### 3.3. Models

Results of the regression model were reported in Table S5. Among the investigated risk factors, only the ethnic group turned out to be significantly associated with body fatness; in particular, African children were almost three times more likely to present overweight (or obesity) compared to Caucasian children (OR = 3.08, 95% CI: 1.29–7.34 in the first model, with almost similar results in the second one). Other ethnic groups (Asian, Hispanic, and mixed) also showed a higher risk. Having a medium adherence to MD was moderately associated (OR = 0.44, 95% CI: 0.17–1.14) with a lower risk of being overweight/obese, while having a high adherence was slightly associated (OR = 0.83, 95% CI: 0.31–2.26); however, this variable was not statistically significant.

### 3.4. Data After Six Months (T1)

#### 3.4.1. Anthropometric Measures

Comparison among anthropometric measures between the baseline and six-month follow-up is shown in Table 4. A non-significant shift in BMI classes was noted, with a slight reduction in children in the obesity and overweight classes in favor of the normal-weight class. Also, according to WHtR, there was a slight reduction in the prevalence of central obesity.

#### 3.4.2. Eating Behaviors and Lifestyle Habits

Table 5A shows the data regarding eating behaviors at T0 and T1 (n = 186). No change was observed at T1 in MD adherence, while improvement, although not significant, in daily hydration was observed, with a higher percentage of children drinking at least 1.5L per day (13.4% vs. 17.2%). However, a decline in the consumption of breakfast and whole cereals emerged. The same consumption preference for ultra-processed foods observed at T0 remained similar at the 6-month follow-up (T1), although with a slight decrease (Table S6).

Table 5B shows the data about lifestyle at T0 and T1 (n = 178). At T1, a favorable trend was observed in the decreasing prevalence of children who did not enjoy any physical activity, while the number of children who enjoy planned physical activity increased. An improvement was also observed in the number of children who slept recommended 9 h or more. On the other hand, screen time seems to have increased, although not significantly.

### 3.5. Educational Intervention Evaluation

#### 3.5.1. Children’s Knowledge Questionnaire

Learning questionnaires have been compiled by children at the beginning of the following school year (T2). All in all, 168 (61.1%) questionnaires out of 275 children attending the participating classes were collected. Results were provided for children who attended at least four out of six lessons (N = 161, 95.83%).

Overall, 85 children (52.8%) achieved a passing score based on the total lesson score, while only 11 children (6.8%) met the sufficiency in every individual topic, with higher prevalence among females in both cases. The prevalence, instead, was lower among children attending the 2nd grade (22.2%), where no children reached the passing score in each topic. Table 6 reports the average score attained in each topic. The most challenging topics for children to grasp were macronutrients and micronutrients, with average scores of 4.07 (SD 2.60) and 3.92 (SD 2.54), respectively. In contrast, the most well-understood topics were the food pyramid, the healthy plate, and lifestyle, all of which had average scores exceeding 7.

#### 3.5.2. Children’s Satisfaction Questionnaires

A total of 244 questionnaires were collected. Overall, 93.9% of children gave a positive evaluation of the educational intervention (question 1, Q1), and 92.1% found the proposed activities interesting and enjoyable (Q2). Additionally, 86.6% considered the language used appropriate for understanding the lesson (Q10), while 90.2% deemed the teaching materials suitable (Q11). On the other hand, 30% of participants reported difficulties in working with their classmates during the activities (Q7). Nevertheless, 89% expressed the desire for the project to be repeated, highlighting a high level of overall satisfaction (Q14). Other than the playful activities, which were the most appreciated by more than half of the sample (n = 134, 54.9%), the most voted lesson was that on the “Food pyramid” (n = 102, 41.8%), while “Micronutrients” was the least appreciated (n = 40, 16.4%). The results are shown in Table S7.

#### 3.5.3. Parents’ Satisfaction Questionnaires

A total of 164 families returned the completed questionnaire. Among them, 75.3% expressed great satisfaction with their child’s participation (Q1), thinking their children improved their knowledge about nutrition and a healthy lifestyle. The results are shown in Table S8.

#### 3.5.4. Teachers’ Satisfaction Questionnaires

The questionnaire for teachers was completed by all participants (n = 18). All teachers (100%) agreed that the educational intervention objectives were well-defined (Q1). Additionally, 94.4% believed that initiatives like this could have a positive impact on public health, beyond benefiting individual children (Q2), and all respondents (100%) considered the proposed activities effective in raising children’s awareness of the importance of a healthy diet (Q13). The language and materials used received positive feedback from 72% of the teachers (Q9). However, some concerns were raised about the educational intervention’s ability to integrate into daily school activities (Q14), with 11% rating this aspect as “low”. Nevertheless, 77.9% expressed the desire for the project to be repeated (Q16). The results are shown in Table S9.

## 4. Discussion

The LIVELY project engaged 14 classes, comprising 275 children, in a multicultural urban primary-school setting, who participated in the educational intervention. Among them, 227 children were included in the data collection to investigate overweight and obesity. Notably, our sample included children from a variety of ethnic backgrounds, most of whom were from Northern Africa and Southern America. This diversity offered both valuable opportunities and unique challenges.

The prevalence of overweight (including obesity) was consistent with national and regional trends [8,9], with the highest rates observed among African and Hispanic children. This finding is in line with the existing literature, which highlights the disproportionately high burden of childhood obesity among ethnic minority groups [50,51,52].

Given BMI limitations in distinguishing fat mass from muscle mass, and variations across age, sex, and ethnic groups, the WHtR was used as a surrogate marker of excess total body adiposity and central adiposity [53,54]. In the overall sample, the prevalence of central obesity was higher than the BMI overweight–obesity classification and showed a significant difference between ethnic groups, with Hispanic and African children showing the highest prevalence compared to Caucasian children.

Notably, this high prevalence of overweight and obesity was often unrecognized by parents, who were more likely to underestimate their child’s weight status, especially if the child was overweight or obese. This is not in contrast with recent studies, where parents misperceived and often underestimated their child’s weight [55,56]. It should be noted that most of the children in our sample had at least one parent who presented as overweight or obese. This may partly explain this misperception, as the literature indicates that parents, particularly mothers, with overweight or obesity are more likely to underestimate their children’s weight status [57]. After six months, a shift from obesity toward a lower BMI category and lower central adiposity was noted; however, these changes were not statistically significant.

Eating behavior analysis revealed that most of the sample exhibited a moderate adherence to the MD. Low intake of vegetables, nuts, and dairy products emerged as the main contributors to reduced adherence levels. These findings are in line with the previous literature identifying insufficient consumption of fruits, vegetables, legumes, and dairy products as key drivers of poor adherence to the MD [58]. No significant correlation between BMI and adherence to the MD was found in our sample, as highlighted in previous studies in which anthropometric measures were more influenced by demographic and social factors rather than adherence to the MD [59,60].

The assessment of lifestyle habits revealed a low adherence to the WHO guidelines for children [38,39], characterized by limited time dedicated to physical activity and nighttime rest, and, conversely, excessive screen time. These are all recognized risk factors for the development of childhood obesity, highlighting the urgent need to implement educational programs that address not only nutrition, but also broader aspects of lifestyle, in order to improve children’s overall quality of life [5,6].

Although there was a positive trend in improved hydration habits and increased physical activity, no significant changes in adherence to the MD were observed after six months. However, these findings are consistent with other studies that have shown limited success in achieving statistically significant changes in childhood obesity prevention over short time periods [61,62].

As highlighted in the literature, an effective school-based nutrition education program requires a balanced integration of formal instruction, practical activities, and active family involvement [5,63,64]. The LIVELY project implemented this multifaceted approach by combining traditional lessons supported by interactive slides and playful activities to enhance message retention. Family engagement was promoted through initiatives such as the creation of a multicultural recipe book and a year-end event, during which families prepared traditional dishes, an occasion that fostered cultural exchange and strengthened participation. Such involvement has been shown to be a key factor in successful childhood obesity prevention programs [65,66].

Similar approaches have been successfully adopted in other school-based interventions, such as the *Feel4Diabetes* program in Europe, which highlighted the importance of family engagement, culturally sensitive materials, and a whole-school approach in effectively promoting healthy behaviors, especially in socioeconomically disadvantaged and ethnically diverse communities [67]. Furthermore, our findings are consistent with those of a randomized trial conducted in Southern Italy [68], where a six-month educational intervention led to a favorable trend in BMI and waist circumference among primary school children, despite the absence of statistically significant differences. This supports the view that short-term interventions may initiate positive change, even if measurable outcomes require longer timeframes.

Finally, children’s knowledge was assessed through a dedicated questionnaire. Overall, the results indicated a moderate level of understanding, with over half of the participants reaching a satisfactory score, and with girls tending to perform better than boys. Notably, the greatest challenges emerged among the younger children, particularly in second grade, where only a small portion of the class met the expected threshold. These findings are consistent with previous research showing that nutritional knowledge tends to increase with age [69], raising important questions about the most appropriate time to introduce nutrition education programs and the most adequate age-targeted approach. For instance, in another project targeting middle school students, a significant improvement in nutritional knowledge was observed in the intervention group [32,35].

This project encountered several challenges that limited its overall effectiveness. First, the failure to reach the planned sample size prevented an accurate analysis of risk factors associated with overweight and obesity. Moreover, a substantial number of children and their families were lost to follow-up at 12 months, potentially introducing attrition bias. This limitation prevented us from drawing reliable conclusions regarding changes in dietary and lifestyle habits or the long-term impact of the intervention. Linguistic, cultural, and social barriers within families might have hindered the completeness of data collection. Additionally, families dealing with weight-related issues may have perceived stigma, which could have contributed to a high discomfort or reluctance in returning the questionnaires, leading to a lower response rate at the second data collection. In fact, a comparison of baseline questionnaire responses revealed that families who did not return the questionnaires at the 12-month follow-up had a higher prevalence of overweight and obesity compared to those who did. This is in line with a previous study that highlighted how body weight can influence individuals’ social identity, and how weight stigma may represent a threat to this identity, leading to concerns about being negatively stereotyped [70]. These barriers also reduced opportunities for broader family engagement and made it difficult to organize meetings with parents, which could have supported and expanded on key topics introduced in class—particularly those related to feeding practices.

On the other hand, the multiethnic setting represented a valuable opportunity for the exchange and integration of diverse habits, dietary patterns, and culinary traditions. These limitations are consistent with those observed in other school-based obesity prevention programs, where language barriers and low parental involvement are commonly reported challenges [71,72].

Second, the intervention was implemented in a single primary school in Northern Italy, without a control group, limiting both the assessment of effectiveness and the generalizability of findings. Nonetheless, the study offers important proof-of-concept evidence for the feasibility, acceptability, and potential impact of school-based nutrition education programs. Indeed, the absence of statistically significant results should not be interpreted as a lack of efficacy but rather as a reflection of the limited duration of the intervention.

Finally, the short-term follow-up may not have been adequate to detect meaningful changes in behavior or anthropometric measures. As suggested by previous research, longer and more intensive interventions are typically needed to induce lasting changes, particularly in children whose habits are still largely shaped by family and living environments [61]. This is in line with evidence from reviews of international school-based obesity prevention programs, which highlight the importance of intervention duration, family involvement, and integration into school culture for sustainable impact [73].

Despite these challenges, the LIVELY project also demonstrated strengths. The educational materials and activities were carefully tailored to be age-appropriate, except for the very youngest children, to ensure that children at different developmental stages could fully engage with the content. The lessons were consistently well attended and highly participatory, with children showing enthusiasm and active involvement throughout the sessions. Additionally, the use of digital tools, such as the interactive lab, was well received by the children, enabling them to learn through play and making educational content more engaging. Furthermore, the project received positive evaluations not only from the children but also from their families, who valued the program for increasing children’s knowledge about nutrition and healthy lifestyles. Most teachers expressed strong interest in repeating the experience in future school years, highlighting the program’s relevance and feasibility within the school context. This encouraging feedback supports that the potential for the project could be adapted and refined for improved implementation and greater impact in future iterations.

## 5. Conclusions and Prospects

The LIVELY project highlights the persistent challenges of addressing childhood obesity within a multicultural, low socio-economic urban school setting. While no significant short-term changes were observed, the study reinforces the importance of schools as key settings for long-term health promotion. To maximize impact, future interventions should be longer and more structured, ideally integrated into the national school curriculum. Additionally, the use of digital tools such as mobile apps, gamified learning modules, and interactive monitoring systems may provide continuous motivation and support beyond the classroom. To fully harness this potential, family engagement must be strengthened, as parents remain the primary influencers of children’s dietary habits at this age. Providing support for families facing linguistic and cultural barriers, potentially through cultural mediators, would have improved participation other than improved overall effectiveness. Furthermore, collaboration with other stakeholders such as pediatricians, psychologists, and family counselors can enhance the intervention’s effectiveness. Overall, embedding culturally tailored, multi-level nutrition education within schools and linked community networks holds promise for sustainable childhood obesity prevention and healthier futures.

## Figures and Tables

**Table 1:** Summary of the lectures and activities proposed during the LIVELY intervention.

Lectures Title	Covered Topics	Games/Activities	Lectures Aim
Macronutrients	Carbohydrates; proteins; lipids; water	True or false; crossword puzzle; game of sets; rhyming riddles	Learn what macronutrients are, their functions, their importance to our bodies, and what foods they are present in. Know the importance of water, its main functions, and daily intake.
Micronutrients	Vitamins and minerals	Rhyming riddles; the hangman’s game; memory game	Learn what micronutrients are, their functions, their importance to our bodies, and what foods they are present in.
Digestive system	The pathway of food from the mouth to the esophagus, to the stomach, to the intestines; the enzymes involved; and where major macronutrients are absorbed	World search; true or false; rhyming riddles; vision of a short cartoon on the digestive system; human anatomy labeling game	Learn what happens to food when we eat, what path it takes inside our bodies, how food is digested, and how nutrients are absorbed.
Food pyramid	Composition of the food pyramid; frequency of food consumption; ultra-processed foods; food labels	Rebus; food pyramid sorting game; riddles	Learn the frequency of food consumption; know the foods to consume every day and those to consume only occasionally. Learn what ultra-processed foods are and learn how to read food labels.
Healthy eating plate	Plate composition; How to compose a healthy dish; importance of breakfast; seasonality of fruits and vegetables	Create your own healthy dish game; Find the Odd One Out game; Find the Missing Part game	Learn how to compose a balanced, healthy dish; learn about alternatives for each part of the dish (fiber, carbohydrates, and protein); learn how to compose a healthy dish on your own by choosing seasonal foods.
Lifestyle	Sleep habits; screen time; physical activity	True or false; riddles; Complete your healthy day activity	Know what the good habits are to stay healthy and lead an active lifestyle; know the difference between moderate and intense physical activity and related examples; learn how much time should be devoted to these activities; know how many hours of sleep a child needs and what are good habits before sleeping; know what the limit of screen time is and what are alternative activities.
Multimedia lab	Drawing and storytelling	Drawing a humanized food and proposing a dramaturgy	To work on the content learned in previous lessons, activate motivation and discussion among students, and work on character design and the creation of shared dramaturgies.

**Table 2:** Distribution of demographic and anthropometric characteristics of 227 children enrolled in the LIVELY study at baseline (T0).

	Males n = 110	Female n = 117	Total n = 227
**Age** (years); mean (SD)	8.9 (1.2)	8.9 (1.1)	8.9 (1.2)
**Place of birth**			
Italy	83 (75.5%)	97 (82.9%)	180 (79.3%)
Africa	10 (9.1%)	13 (11.1%)	23 (10.1%)
South America	6 (5.5%)	5 (4.3%)	11 (4.8%)
Europe	9 (8.2%)	1 (0.9%)	10 (4.4%)
Asia	2 (1.8%)	1 (0.9%)	3 (1.3%)
**Attended class**			
Second grade (6–7 years old)	8 (7.3%)	4 (3.4%)	12 (5.3%)
Third grade (7–9 years old)	36 (32.7%)	41 (35.0%)	77 (33.9%)
Fourth grade (8–10 years old)	17 (15.5%)	19 (16.2%)	36 (15.9%)
Fifth grade (9–12 years old)	49 (44.5%)	53 (45.3%)	102 (4.9%)
**Ethnicity**			
Caucasian	44 (40.0%)	38 (32.5%)	82 (36.1%)
African	36 (32.7%)	50 (42.7%)	86 (37.8%)
Hispanic	16 (14.5%)	15 (12.8%)	31 (13.7%)
Asian	9 (8.2%)	11 (9.4%)	20 (8.8%)
Mixed	5 (4.6%)	3 (2.6%)	8 (3.5%)
**Anthropometric measurements**			
Weight, kg; mean (SD)	35.27 (10.5)	36.41 (10.57)	35.86 (10.53)
Height, cm; mean (SD)	135.85 (8.38)	136.75 (9.09)	136.33 (8.74)
Waist circumference, cm; mean (SD)	65.22 (10.95)	65.15 (11.79)	65.19 (11.37)
**BMI z-score classes**			
Underweight	4 (3.6%)	1 (0.9%)	5 (2.2%)
Normal weight	80 (72.7%)	89 (76.1%)	169 (74.4%)
Overweight	16 (14.5%)	24 (21.4%)	41 (18.1%)
Obesity	10 (9.1%)	3 (1.7%)	12 (5.3%)
**WHtR**			
WHtR < 0.5	76 (69.1%)	74 (63.2%)	150 (66.1%)
WHtR ≥ 0.5	34 (30.9%)	43 (36.8%)	77 (33.9%)

Data are expressed as counts (%) unless otherwise specified; Body Mass Index (BMI); Waist-to-Height ratio (WHtR); Standard deviation (SD).

**Table 3:** Children’s eating behaviors and lifestyle habits at baseline (T0).

	Total Sample N = 205	
	N (%)	Missing
**Adherence to Mediterranean Diet**		
Low	29 (14.4%)	4
Medium	111 (55.2%)	
High	61 (30.4%)	
**Breakfast consumption**	160 (78.4%)	1
**Consumption of whole foods** *(pasta, cereals, bread, breakfast cereals%)*	130 (64.4%)	3
**Use of sweeteners** *(sugar, honey, sweetened cocoa,* etc.*%)*	143 (70.1%)	1
**Hydration**		
<1 L a day	69 (33.8%)	1
1–1.5 L a day	108 (52.9%)	
>1.5 L a day	27 (13.3%)	
**Physical activity**		
No physical activity	18 (9.0%)	4
Only non-programmed physical activity	50 (24.9%)	
1–2 times a week of programmed physical activity	93 (46.3%)	
3 or more times a week of programmed physical activity	40 (19.9%)	
**Screen time**		
~2 h or less	175 (89.3%)	9
~3 h or more	21 (10.7%)	
**Sleep hygiene**		
Less than 9 h of sleep	66 (33.3%)	7
9 or more hours of sleep	132 (66.7%)	

Categorical variables are expressed as counts (percentages).

**Table 4:** Children’s anthropometric measures at baseline (T0) and at 6 months follow-up (T1).

	Total Sample n = 194	
	Baseline (T0)	Six Months (T1)
**Anthropometric measurements**		
Weight, kg; mean (SD)	35.91 (10.06)	37.53 (10.86)
Height, cm; mean (SD)	136.49 (8.40)	139.91 (8.73)
Waist circumference, cm; mean (SD)	65.24 (11.07)	65.47 (11.44)
**BMI z-score classes**		
Underweight	4 (2.1%)	6 (3.1%)
Normal weight	144 (74.2%)	149 (76.8%)
Overweight	36 (18.6%)	30 (15.5%)
Obesity	10 (5.1%)	9 (4.6%)
**WHtR**		
WHtR < 0.5	130 (67.0%)	143 (73.7%)
WHtR ≥ 0.5	64 (33.0%)	51 (26.3%)

Data are expressed as counts (%) unless otherwise specified; Body Mass Index (BMI); Waist-to-Height ratio (WHtR); Standard deviation (SD).

**Table 5:** Children’s eating behaviors and lifestyle habits (T0) and at 6 months follow-up (T1). (**A**) Eating behaviors. (**B**) Lifestyle habits.

A		
	Total Sample n = 186	
	Baseline (T0)	Six Months (T1)
**Adherence to Mediterranean Diet**		
Low	27 (14.5%)	30 (16.1%)
Medium	103 (55.4%)	98 (52.7%)
High	56 (30.1%)	58 (31.2%)
**Breakfast consumption**	145 (79.0%)	125 (67.2%)
**Consumption of whole foods** *(pasta, cereals, bread, breakfast cereals)*	116 (62.4%)	109 (58.6%)
**Use of sweeteners** *(sugar, honey, sweetened cocoa, etc.)*	128 (68.8%)	127 (68.3%)
**Hydration**		
<1 L a day	61 (32.8%)	60 (32.3%)
1–1.5 L a day	100 (53.8%)	94 (50.5%)
>1.5 L a day	25 (13.4%)	32 (17.2%)
**B**		
	**Total sample** **n = 178**	
	**Baseline (T0)**	**Six months (T1)**
**Physical activity**		
No physical activity	15 (8.4%)	10 (5.6%)
Only non-programmed physical activity	41 (23.0%)	36 (20.2%)
1–2 times a week of programmed physical activity	86 (48.3%)	89 (50.0%)
3 or more times a week of programmed physical activity	36 (20.2%)	43 (24.2%)
**Screen time**		
~2 h or less	161 (90.4%)	163 (86.5%)
~3 h or more	17 (9.6%)	25 (13.5%)
**Sleep hygiene**		
Less than 9 h of sleep	57 (32.0%)	48 (27.0%)
9 or more hours of sleep	121 (68.0%)	130 (73.0%)

Categorical variables are expressed as counts (percentages).

**Table 6:** Average scores (SD) obtained in the knowledge questionnaire.

Topic	Average Score
Macronutrients	4.07 (2.60)
Micronutrients	3.92 (2.54)
Digestion	6.71 (1.72)
Food pyramid	7.09 (2.26)
Healthy plate	7.24 (3.31)
Lifestyle	7.22 (2.99)
Total	6.04 (1.50)

Data are expressed as mean (SD); Standard deviation (SD).

## Data Availability

The data that support the findings of this study are available from the corresponding author upon reasonable request, subject to prior authorization from the Istituto di Ricerche Farmacologiche Mario Negri IRCCS Milan, the Data Controller in accordance with GDPR. Data sharing is restricted to comply with the regulations of the European Economic Area (EEA). As such, data sharing with entities or individuals located outside the EEA is not foreseen.

## Supplementary Materials

The following supporting information can be downloaded at: https://www.mdpi.com/article/10.3390/nu17172778/s1. Table S1: Children’s anthropometric measures distributed by ethnicity; Table S2: Mothers’ and fathers’ characteristics; Table S3. Distribution of answers at each KIDMED questions at baseline (T0); Table S4: Frequency of children who often (≥4 time a week) eat ultra-processed foods at baseline (T0); Table S5: Logistic regression model of association of overweight/obesity with nutritional factors (Model 1) and lifestyle (Model 2); Table S6: Frequency of children who often (≥4 time a week) eat ultra-processed foods at baseline (T0) and at 6 months follow-up (T1); Table S7: Responses to the satisfaction questionnaire addressed to children; Table S8: Responses to the satisfaction questionnaire addressed to parents or caregivers; Table S9: Responses to the satisfaction questionnaire addressed to teachers; Figure S1: Summary of the topics covered during Lecture n° 2 “Micronutrients”; Figure S2: Laminated placemat.

## Abbreviations

The following abbreviations are used in this manuscript:

BMI	Body Mass Index
COSI	Childhood Obesity Surveillance Initiative
CREA	Centro di Ricerca Alimenti e Nutrizione
CVD	Cardiovascular disease
KIDMED	Mediterranean Diet Quality Index for children and adolescents
IMN	Istituto di Ricerche Farmacologiche Mario Negri IRCCS (IMN)
LIVELY	MuLtidimensional school-based and family Involved interVentions, to promote a hEalthy and sustainable LifestYle for the childhood obesity primary prevention
LDNC	Laboratory of Dietetics and Clinical Nutrition
MD	Mediterranean Diet
NCDs	Non communicable diseases
SD	Standard deviation
WC	Waist circumference
WHO	World Health Organization
WHtR	Waist-to-height ratio
UNESCO	United Nations Educational, Scientific and Cultural Organization
