# Supplementary material for: Lights and Shadows of a Primary School-Based Nutrition Education Program in Italy: Insights from the LIVELY Project
Source: Nutrients. 2025 Aug 27;17(17):2778. doi: 10.3390/nu17172778 (PMC12430468; doi:10.3390/nu17172778)
Supplement: Supplementary file 1 [file nutrients-17-02778-s001.zip › nutrients-3817513-supplementary.pdf]

nutrients

MDPI

Supplementary material for "Lights and Shadows of a Primary School-Based Nutrition Education Program in Italy: Insights from the LIVELY Project"

Supplementary data for baseline (T0) data collection

Table S1. Children's anthropometric measures distributed by ethnicity

	Total sample n=227				
	**Caucasic N=82**	**African N=86**	**Hispanic N=31**	**Asian N=20**	**Mixed N=8**
**BMI z-score classes**					
Underweight	1 (1.2%)	1 (1.2%)	0 (0.0%)	3 (15.0%)	0 (0.0%)
Normal weight	70 (85.4%)	57 (66.3%)	21 (67.8%)	14 (70.0%)	7 (87.5%)
Overweight	6 (7.3%)	21 (24.4%)	9 (29.0%)	3 (15.0%)	1 (12.5%)
Obesity	5 (6.1%)	7 (8.1%)	1 (3.2%)	0 (0.0%)	0 (0.0%)
**WHtR**					
WHtR<0.5	62 (75.6%)	55 (64.0%)	13 (41.9)	13 (65.0%)	7 (87.5%)
WHtRε0.5	20 (24.4%)	31 (36.0%)	18 (58.1%)	7 (35.0%)	1 (12.5%)

Data are expressed as counts (%) unless otherwise specified. Body Mass Index (BMI); Waist to Height ratio (WHtR).

Table S2. Mothers' and fathers' characteristics

	**Mothers n=205**	**Fathers n=190**
**Study title**		
No one	11 (5.5%)	10 (5.6%)
Elementary school license	9 (4.5%)	9 (5.0%)
Middle school license	37 (18.4%)	33 (18.4%)
High school license	74 (36.8%)	70 (39.1%)
University degree or above	70 (34.8%)	57 (31.8%)
Missing	4	11
**Occupation**		
Yes	125 (70.2%)	162 (98.8%)
No	53 (29.8%)	2 (1.2%)
Missing	27	26
**BMI class**		
Underweight	5 (2.6%)	3 (1.8%)
Normal weight	83 (43.0%)	64 (37.9%)
Overweight	71 (37.8%)	85 (50.3%)
Obesity	34 (17.6%)	17 (10.0%)
Missing	12	11
**Physical activity**		
Yes	134 (70.2%)	117 (64.6%)
No	57 (29.8%)	64 (35.4%)
Missing	14	9

Categorical variables are expressed as counts (percentages).

Table S3. Children's eating behaviors and lifestyle habits at baseline (T0)

	Total sample N=205	
	**N (%)**	**Missing**
**Adherence to Mediterranean Diet**		
Low	29 (14.4%)	4
Medium	111 (55.2%)	
High	61 (30.4%)	
**Breakfast consumption**	160 (78.4%)	1
**Consumption of whole foods** *(pasta, cereals, bread, breakfast cereals%)*	130 (64.4%)	3
**Use of sweeteners** *(sugar, honey, sweetened cocoa etc.%)*	143 (70.1%)	1
**Hydration**		
< 1L a day	69 (33.8%)	1
1L – 1.5L a day	108 (52.9%)	
> 1.5 L a day	27 (13.3%)	
**Physical activity**		
No physical activity	18 (9.0%)	4
Only non-programmed physical activity	50 (24.9%)	
1-2 times a week of programmed physical activity	93 (46.3%)	
3 or more times a week of programmed physical activity	40 (19.9%)	
**Screen time**		
~ 2 h or less	175 (89.3%)	9
~ 3 h or more	21 (10.7%)	
**Sleep hygiene**		
Less than 9 hours of sleep	66 (33.3%)	7
More than 9 hours of sleep	132 (66.7%)	

Categorical variables are expressed as counts (percentages).

Table S4. Distribution of answers at each KIDMED questions at baseline (T0)

	Total sample N=204		
	**Yes**	**No**	**Missing**
**KIDMED questions**			
Does the child consume a fruit or a juice every day?	168 (82.4%)	36 (17.6%)	0
Does the child consume a second fruit every day?	113 (56.2%)	88 (43.8%)	3
Does the child consume vegetables, cooked or raw, regularly, once a day?	146 (71.9%)	57 (28.1%)	1
Does the child consume vegetables, cooked or raw, more than once a day?	78 (39.0%)	122 (61.0%)	4
Does the child regularly consume fish (at least 2-3 times a week)?	93 (46.0%)	109 (54.0%)	2
Does the child go to fast food restaurants (hamburgers) more than once a week?	37 (18.1%)	167 (81.9%)	0
Does the child like legumes and consume them more than once a week?	122 (59.8%)	82 (40.2%)	0
Does the child consume pasta and rice every day (5 or more times a week)?	158 (77.5%)	46 (22.5%)	0
Does the child consume whole grains or bread for breakfast?	108 (54.3%)	91 (45.7%)	5
Does the child regularly consume nuts (at least 2-3 times a week)?	64 (31.7%)	138 (68.3%)	2
Is olive oil used at home for preparing the child's meals?	180 (90.0%)	20 (10.0%)	4
Does the child skip breakfast?	51 (25.1%)	152 (74.9%)	1
Does the child consume dairy products for breakfast (yogurt, milk, etc.)?	165 (81.3%)	38 (18.7%)	1
Does the child consume baked products or pastries for breakfast?	89 (44.1%)	113 (55.9%)	2
Does the child consume two yogurts and/or 40g of cheese daily?	61 (30.4%)	140 (69.6%)	3
Does the child consume candies or sweets multiple times a day?	54 (26.7%)	148 (73.3%)	2

Categorical variables are expressed as counts (percentages).

Table S5. Frequency of children who often (≥4 time a week) eat ultra-processed foods at baseline (T0), at 6 months follow-up (T1) and at 12 months follow-up (T2)

	**Total sample N=100**					
	**Baseline (T0)**		**Six months (T1)**		**Twelve months (T2)**	
	N (%)	Missing	N (%)	Missing	N (%)	Missing
**Ultra-processed food**						
Biscuits	43 (43.0%)	-	40 (40.4%)	1	38 (38.4%)	1
Milk-based beverages	35 (35.4%)	1	38 (38.0%)	-	35 (36.5%)	4
Fruits juices	27 (27.3%)	1	23 (23.2%)	1	24 (24.5%)	2
Industrial bread	21 (21.2%)	1	19 (19.0%)	-	15 (15.2%)	1
Salty snack	18 (18.0%)	-	11 (11.2%)	2	10 (10.1%)	1
Sweets and cake	17 (17.0%)	-	13 (13.3%)	2	17 (17.0%)	-
Breakfast cereals	16 (16.0%)	-	21 (21.7%)	3	19 (19.2%)	1
Sausages and other meat products	10 (10.1%)	1	16 (16.3%)	2	18 (18.4%)	2
Industrial dessert	8 (8.0%)	-	9 (9.0%)	1	8 (8.1%)	1
Gravy, condiments and ready-to-eat sauce	7 (7.1%)	1	12 (12.1%)	1	10 (10.1%)	1
Chips	5 (5.0%)	-	4 (4.0%)	1	5 (5.1%)	1
Confetti	4 (4.1%)	2	6 (6.0%)	-	7 (7.0%)	-
Margarine and other creams	3 (3.0%)	-	5 (5.1%)	2	6 (6.1%)	1
Confectioned ready-to-eat meal	2 (2.0%)	-	1 (1.0%)	-	5 (5.1%)	1
Soy and other beverages substituted of milk	2 (2.0%)	2	2 (2.0%)	1	3 (3.0%)	1
Industrial pizza	1 (1.0%)	-	1 (1.0%)	1	2 (2.0%)	1

Categorical variables are expressed as counts (percentages).

Table S6. Odds Ratios (OR) and 95% Confidence Interval (95% CI) from logistic regression model of association of overweight/obesity with nutritional factors (Model 1) and lifestyle (Model 2)

	**Model 1***	**Model 2****
**Sex**		
	1.00	1.00
Males	1.03 (0.51-2.06)	1.06 (0.53-2.14)
**Ethnic group**		
Caucasian	1.00	1.00
African	3.08 (1.29-7.34)	3.24 (1.31-8.02)
Others §	2.56 (0.97-6.75)	2.20 (0.80-6.04)
**Mediterranean Diet Adherence**		
Low		
Medium	0.44 (0.17-1.14)	-
High	0.83 (0.31-2.26)	-
**Hydration level**		
< 1 L		
1-1.5 L	0.94 (0.44-2.02)	
>1.5 L	1.46 (0.51-4.15)	
**Physical Activity**		
None		
Not programmed	-	2.69 (0.30-23.75)
Programmed (1-2 times/week)	-	5.46 (0.67-44.24)
Programmed (≥3 times/week)	-	5.82 (0.67-50.39)
**Screen time**		
**(~ 3 h or more)**	-	1.37 (0.68-2.78)
**Sleep hygiene**		
**(≥9 hours)**	-	0.97 (0.45-2.09)

* Model 1 was fitted using 200 observation (46 overweight/obese)

** Model 2 was fitted using 194 observations (43 overweight/obese)

§ Others categories included Asiatic, Hispanic and Mixed ethnic groups.

Supplementary data for the project evaluation

Table S7. Responses to the satisfaction questionnaire addressed to children

Questions	Very	Enough	A little	Not at all	Missing data
1.Did you enjoy participating in this project?	144 (59.3%)	84 (34.6%)	13 (5.4%)	2 (0.8%)	1
2. Do you think participating in this project was useful?	136 (57.1%)	75 (31.5%)	22 (9.2%)	5 (2.1%)	6
3. Do you think the proposed activities helped you understand the importance of a healthy diet?	142 (58.7%)	85 (35.1%)	12 (5.0%)	3 (1.2%)	2
4. Do you think the proposed activities helped you understand the importance of a healthy lifestyle?	133 (55.0%)	89 (36.8%)	14 (5.8%)	6 (2.5%)	2
5. Do you think the number of meetings we had was sufficient?	93 (38.9%)	87 (36.4%)	41 (14.2%)	18 (7.5%)	5
6. Did you feel engaged in the proposed activities?	114 (47.7%)	83 (34.7%)	38 (15.9%)	4 (1.7%)	5
7. Did you have any difficulties working with your classmates in the group?	27 (11.4%)	44 (18.6%)	67 (28.4%)	98 (41.5%)	8
8. Do you think the proposed activities were interesting/fun?	146 (60.8%)	75 (31.3%)	13 (5.4%)	6 (2.5%)	4
9. Did you understand the topics covered?	73 (30.5%)	135 (56.5%)	31 (13.0%)	-	5
10. Was the language used simple?	118 (49.4%)	89 (37.2%)	27 (11.3%)	5 (2.1%)	5
11. Was the material used easy to understand?	144 (61.0%)	69 (29.2%)	16 (6.8%)	7 (3.0%)	8
12. While doing the activities, did you experience positive emotions (interest, surprise, joy, happiness, fun…%)?	135 (57.9%)	60 (25.8%)	26 (11.2%)	12 (5.2%)	11
13. Did you learn new things?	155 (65.4%)	60 (25.3%)	19 (8.0%)	3 (1.3%)	7

	Yes	No	Missing
14. Would you like the project to be repeated?	209 (89%)	26 (11.0%)	9

Data are expressed as counts (percentages).

Table S8. Responses to the satisfaction questionnaire addressed to parents or caregivers.

Questions	Very	Enough	A little	Not at all	Missing data
1. Are you satisfied that your son/daughter participated in this project?	122 (75.3%)	35 (21.6%)	2 (1.2%)	3 (1.9%)	2
2. Do you think your son/daughter is satisfied that he/she participated in this project?	115 (72.9%)	42 (26.3%)	2 (1.3%)	2 (1.2%)	3
3. In your opinion, was the project helpful in improving your son/daughter's knowledge of proper nutrition and lifestyle?	121 (75.6%)	31 (19.4%)	6 (3.8%)	2 (1.3%)	4

Data are expressed as counts (percentages).

Table S9. Responses to the satisfaction questionnaire addressed to teachers.

Questions	Very	Enough	A little	Not at all
1.In your opinion, were the project objectives well defined?	9 (50%)	9 (50%)	-	-
2. Do you believe that projects like this can have a positive impact on public health, beyond benefiting the individual child?	15 (83.3%)	2 (11.1%)	1 (5.6%)	-
3. Were the content of the sessions and the proposed activities aligned with the project objectives?	13 (72.2%)	4 (22.2%)	1 (5.6%)	-
4. To what extent did the various aspects of the project meet your expectations?	13 (72.2%)	4 (22.2%)	1 (5.6%)	-
5. In your opinion, were the proposed activities presented and conducted in a way that encouraged the engagement and participation of all students?	10 (55.6%)	7 (38.9%)	1 (5.6%)	-
6. Was the overall duration of the project appropriate to achieve its objectives?	12 (66.7%)	5 (27.8%)	1 (5.6%)	-
7. Do you think the project was well-organized and balanced in terms of the educational activities proposed for the class?	6 (33.3%)	9 (50%)	3 (16.7%)	-
8. In your opinion, were the project objectives achieved?	11 (61.1%)	5 (27.8%)	2 (11.1%)	-
9. Do you think the language used by the research team was clear and effective (keeping students engaged%) in relation to their age?	13 (72.2%)	3 (16.7%)	2 (11.1%)	-
10. In your opinion, did the students participate in the proposed activities with enthusiasm?	12 (66.7%)	5 (27.8%)	1 (5.6%)	-
11. Do you think the students actively and constructively collaborated with each other and with the researchers to achieve the project objectives?	6 (33.3%)	11 (61.1%)	1 (5.6%)	-
12. Do you believe the project contributed to students’ learning or improved their knowledge about adopting a healthy diet?	9 (50%)	7 (38.9%)	2 (11.1%)	-
13. In your opinion, were the proposed activities effective in raising children's awareness of the importance of a healthy diet?	8 (44.4%)	10 (55.6%)	-	-
14. Do you think the proposed activities could be integrated into students' daily school routines?	5 (27.8%)	11 (61.1%)	2 (11.1%)	-
15. In your opinion, did the proposed activities increase students' awareness of the importance of a healthy diet and an appropriate lifestyle?	10 (55.6%)	7 (38.9%)	1 (5.6%)	-

	Yes	No	Missing
16. Would you like the project to be repeated?	14 (77.8%)	1 (5.6%)	3 (16.7%)

Data are expressed as counts (percentages

Figure S1. Summary of the topics covered during Lecture n°2 "Micronutrients"

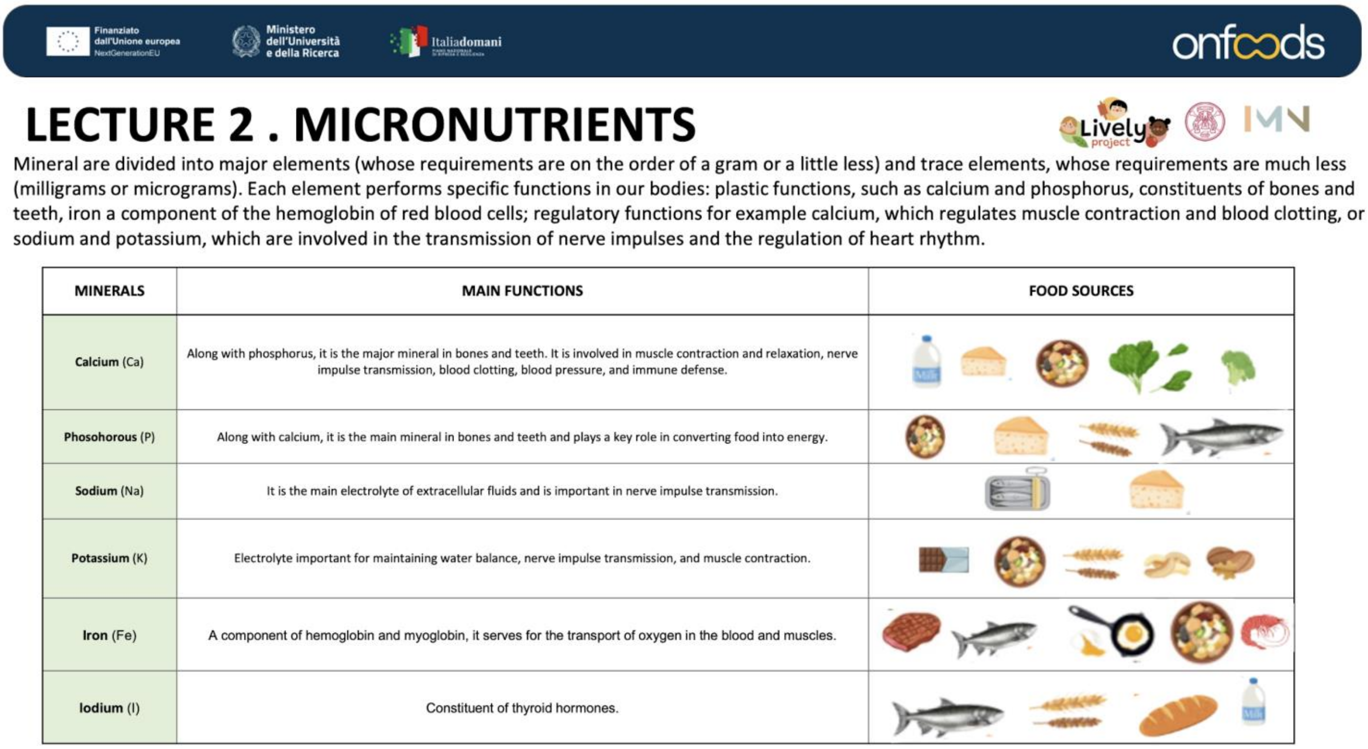

LECTURE 2 . MICRONUTRIENTS

Mineral are divided into major elements (whose requirements are on the order of a gram or a little less) and trace elements, whose requirements are much less (milligrams or micrograms). Each element performs specific functions in our bodies: plastic functions, such as calcium and phosphorus, constituents of bones and teeth, iron a component of the hemoglobin of red blood cells; regulatory functions for example calcium, which regulates muscle contraction and blood clotting, or sodium and potassium, which are involved in the transmission of nerve impulses and the regulation of heart rhythm.

MINERALS	MAIN FUNCTIONS	FOOD SOURCES
**Calcium** (Ca)	Along with phosphorus, it is the major mineral in bones and teeth. It is involved in muscle contraction and relaxation, nerve impulse transmission, blood clotting, blood pressure, and immune defense.	
**Phosohorous** (P)	Along with calcium, it is the main mineral in bones and teeth and plays a key role in converting food into energy.	
**Sodium** (Na)	It is the main electrolyte of extracellular fluids and is important in nerve impulse transmission.	
**Potassium** (K)	Electrolyte important for maintaining water balance, nerve impulse transmission, and muscle contraction.	
**Iron** (Fe)	A component of hemoglobin and myoglobin, it serves for the transport of oxygen in the blood and muscles.	
**Iodium** (I)	Constituent of thyroid hormones.	

Figure S2. Laminated placemat

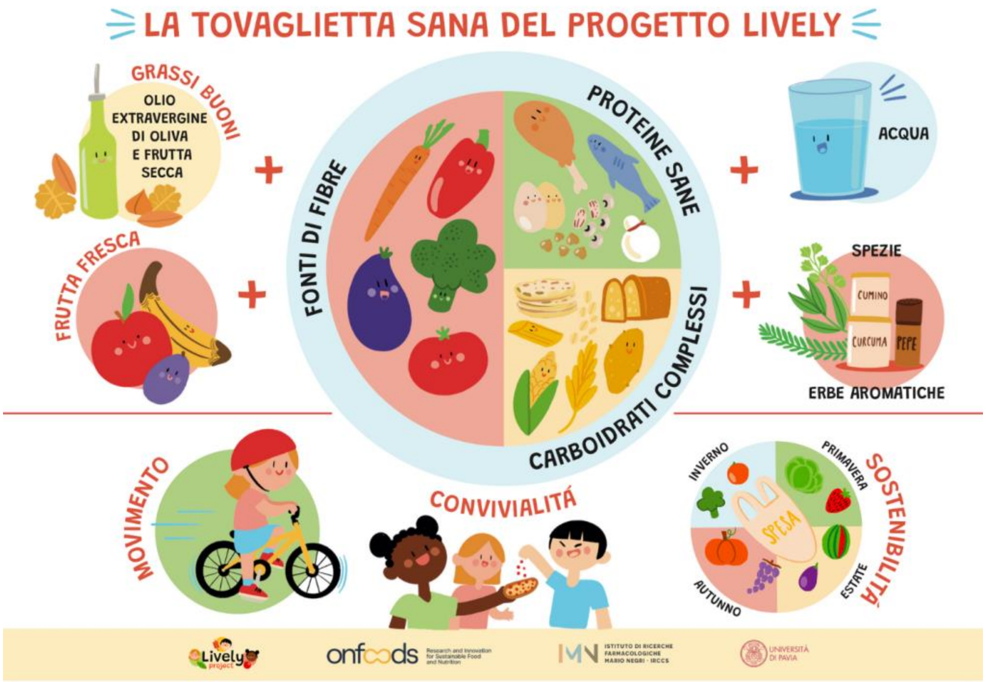

Figure S2A

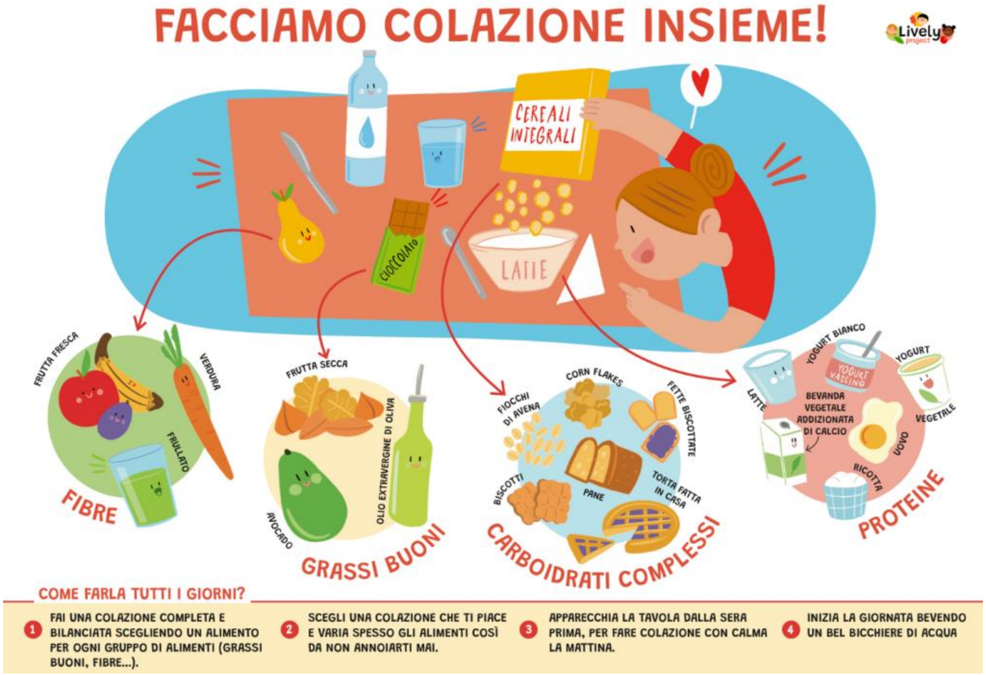

Figure S2B
